# Farmer surveys in Europe suggest that specialized, intensive farms were more likely to perceive negative impacts from COVID-19

**DOI:** 10.1007/s13593-022-00820-5

**Published:** 2022-08-23

**Authors:** Julian Helfenstein, Matthias Bürgi, Niels Debonne, Thymios Dimopoulos, Vasco Diogo, Wenche Dramstad, Anna Edlinger, Maria Garcia-Martin, Józef Hernik, Thanasis Kizos, Angela Lausch, Christian Levers, Franziska Mohr, Gerardo Moreno, Robert Pazur, Michael Siegrist, Rebecca Swart, Claudine Thenail, Peter H. Verburg, Tim G Williams, Anita Zarina, Felix Herzog

**Affiliations:** 1 Agroecology and Environment, Agroscope, Zürich, Switzerland; 2 Institute of Geography, University of Bern, Bern, Switzerland; 3 Land Change Science Research Unit, Swiss Federal Research Institute WSL, Birmensdorf, Switzerland; 4 Environmental Geography Group, Institute for Environmental Studies (IVM), Vrije Universiteit Amsterdam, Amsterdam, The Netherlands; 5 Mediterranean Institute for Nature and Anthropos, MedINA, Athens, Greece; 6 Norwegian Institute of Bioeconomy Research (NIBIO), Ås, Norway; 7 Department of Land management and Landscape Architecture, University of Agriculture in Krakow, Krakow, Poland; 8 Department of Geography, University of the Aegean, Mytilene, Greece; 9 Department Computational Landscape Ecology, Helmholtz Centre for Environmental Research-UFZ, Leipzig, Germany; 10 Geography Department, Humboldt University Berlin, Berlin, Germany; 11 Forest Research Group, University of Extremadura, Plasencia, Spain; 12 Institute of Geography, Slovak Academy of Sciences, Bratislava, Slovakia; 13 Department of Health Sciences and Technology, ETH Zürich, Zürich, Switzerland; 14 INRAe, 35042 Rennes, France; 15 Department of Geography, University of Latvia, Riga, Latvia

**Keywords:** Farmer livelihood, Pandemic, Agriculture, Food system, Land use intensity, Diversification, Resilience

## Abstract

It has been shown that the COVID-19 pandemic affected some agricultural systems more than others, and even within geographic regions, not all farms were affected to the same extent. To build resilience of agricultural systems to future shocks, it is key to understand which farms were affected and why. In this study, we examined farmers’ perceived robustness to COVID-19, a key resilience capacity. We conducted standardized farmer interviews (*n* = 257) in 15 case study areas across Europe, covering a large range of socio-ecological contexts and farm types. Interviews targeted perceived livelihood impacts of the COVID-19 pandemic on productivity, sales, price, labor availability, and supply chains in 2020, as well as farm(er) characteristics and farm management. Our study corroborates earlier evidence that most farms were not or only slightly affected by the first wave(s) of the pandemic in 2020, and that impacts varied widely by study region. However, a significant minority of farmers across Europe reported that the pandemic was “the worst crisis in a lifetime” (3%) or “the worst crisis in a decade” (7%). Statistical analysis showed that more specialized and intensive farms were more likely to have perceived negative impacts. From a societal perspective, this suggests that highly specialized, intensive farms face higher vulnerability to shocks that affect regional to global supply chains. Supporting farmers in the diversification of their production systems while decreasing dependence on service suppliers and supply chain actors may increase their robustness to future disruptions.

**Supplementary Information:**

The online version contains supplementary material available at 10.1007/s13593-022-00820-5.

## Introduction

The early phase of the COVID-19 pandemic severely challenged global food security due to increased food demand as a result of consumer panic buying, disruption of food deliveries and agri-food inputs, labor shortages caused by mobility restrictions, and a slowdown in food production because of virus outbreaks in processing plants (Torero 2020; UN 2020; Montanari et al. 2021). However, supply chains recovered quickly in most areas of the world, leading to overall milder consequences of the pandemic on agricultural production and food supply than initially feared (Snow et al. 2021; Weersink et al. 2021; Zhan and Chen 2021). Yet, the full impact of the pandemic on farmer livelihoods, especially conditions that fostered farmers’ resilience, is largely unknown.

In Europe, although the value of output by the agricultural industry declined by only 1.4% in 2020 compared to 2019, farm incomes declined by 7.9% (Montanari et al. 2021). Such impacts were not evenly distributed across regions or food systems, for example disproportionately affecting those with a high concentration of farms catering to restaurants, which were largely closed in 2020 (Meuwissen et al. 2021). At the farm level, surveys have shown that even within a food system, individual farms were differently impacted (Coopmans et al. 2021; Perrin and Martin 2021; Vargas et al. 2021). For example, a survey of French organic dairy cattle farmers showed that of 86 farmers, 44% were not impacted, 50% faced minor challenges, and 6% faced major challenges (Perrin and Martin 2021). However, only few studies have quantitatively analyzed which farm attributes explain the magnitude of COVID-19 impacts (Coopmans et al. 2021).

Robustness is the capacity to absorb the effects of shocks while retaining basic structure and functioning (Walker and Salt 2006; Béné et al. 2012; Folke 2016), and thus necessary for agricultural systems to achieve societal goals around food security and rural vitality (Darnhofer 2014; Scown et al. 2019). Together with adaptability and transformability, robustness is one of the three resilience capacities (Meuwissen et al. 2019). Many attributes have been hypothesized to foster resilience, ranging from farm-level endowments (e.g., natural and human capital) to larger-scale institutional structures and networks (Ifejika Speranza et al. 2014). In a recent review, Meuwissen et al. (2019) point out that diversity, openness, tightness of feedbacks, modularity, and system reserves all enhance resilience of farming systems. The COVID-19 pandemic, in particular, was a large-scale and unanticipated shock to supply chains and other food system components. While measuring adaptability and transformability will only be possible several years after the pandemic, we can already study the patterns of farmers’ robustness to this shock, as a useful indication of resilience to other future shocks (Stephens et al. 2020).

Resilience capacities can be measured using both objective and subjective indicators (Jones 2019). Objective indicators, such as changes in asset stocks or income, dominate many discussions of resilience as they are conceptually easy to understand and agree with prevailing positivist epistemologies. Subjective indicators, in contrast, focus on people’s self-assessed capacity to deal with risks (Jones 2019). Such perceptions of resilience reflect people’s lived experiences and self-efficacy (Béné et al. 2019), and have been argued by many to add value to objective indicators, for instance because they facilitate cross-cultural comparisons (Clare et al. 2017). In addition to cultural and psychosocial factors, there is evidence that subjective resilience is associated with risk management (Slijper et al. 2020). For instance, pre-COVID-19 farm surveys in several regions across Europe have shown that farmers with higher perceived resilience were younger, had bigger farms, and conducted less labor-intensive farming, among other attributes (Spiegel et al. 2021).

**Fig. 1:**
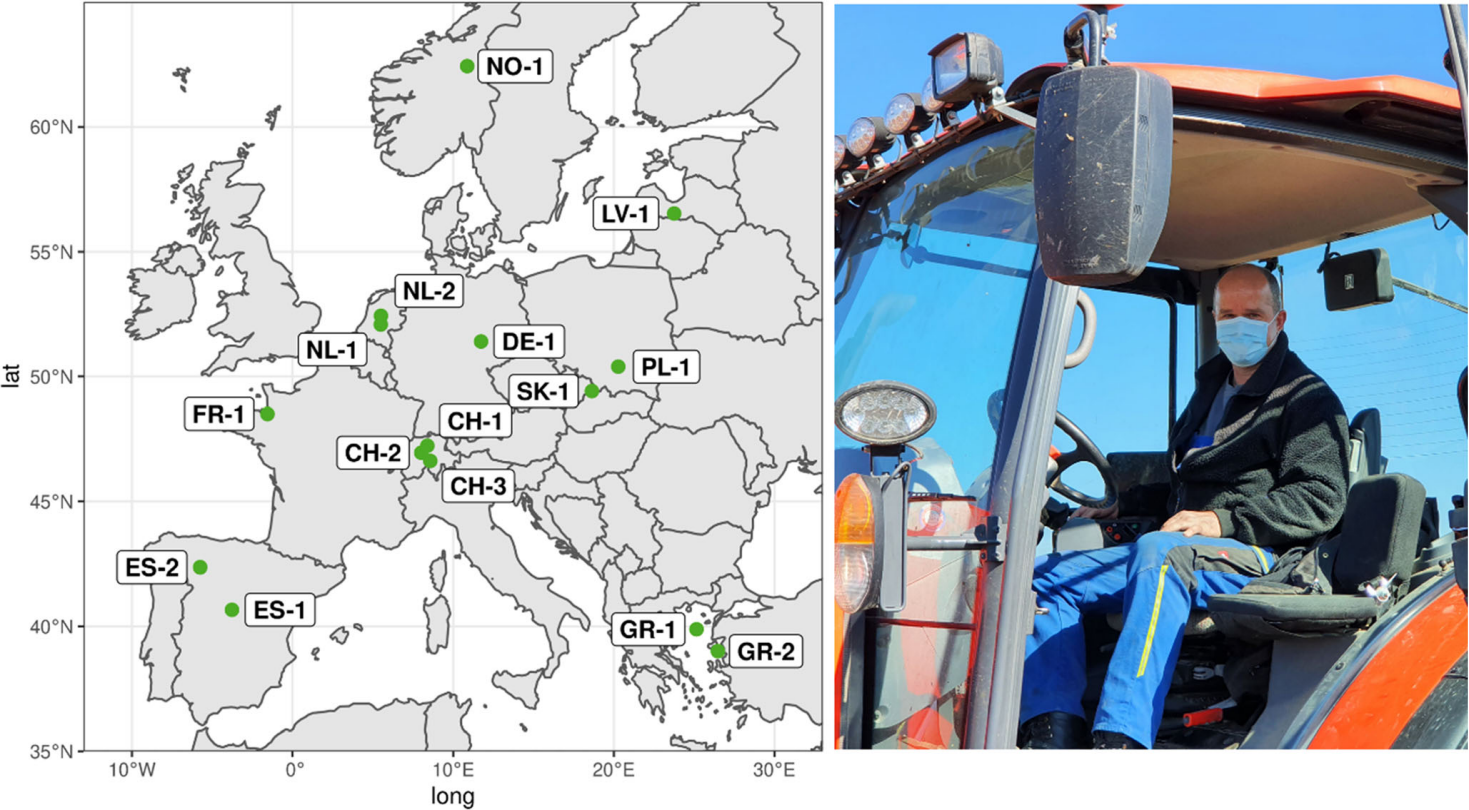
Location of the 15 study sites across Europe where we surveyed the impact of the COVID-19 pandemic on farms. Photograph on the right by Julian Helfenstein.

In this study, we complement objective resilience indicators showing the economic impact of the COVID-19 pandemic on agriculture in Europe (e.g., Montanari et al. 2021) by focusing on subjective robustness as perceived by farmers. We asked farmers in 15 distinct agricultural systems across Europe to self-assess how they were impacted by COVID-19 in the year 2020 (Fig. 1). We then analyzed how the perceived impacts relate with different farm attributes. Specifically, we tested three hypotheses. First, we hypothesized that farms with a lower diversity of products and incomes were more likely to perceive negative impacts (H1). Second, we hypothesized that intensive farms were more likely to perceive negative effects than less intensive farms (H2). Third, we also tested the relationship between farm size and perceived COVID-19 impact. Following Spiegel et al. (2021), we hypothesized that smaller farms perceived to be more affected than bigger farms (H3).

## Methods

### Study sites

The 15 study sites were selected to cover a broad range of agricultural contexts in Europe (Table 1). Eight sites were in primarily arable farming regions (CH-1, DE-1, ES-2, FR-1, GR-1, LV-1, NL-2, PL-1), six in grassland regions (CH-2, CH-3, ES-1, NL-1, NO-1, SK-1), and one was in a permanent crop region (olive farming, GR-2). Dairy and other livestock productions played a major role in 11 out of 15 sites (CH-1, CH-2, CH-3, DE-1, ES-1, FR-1, GR-1, NL-1, NL-2, NO-1, SK-1). This reflects the nature of agriculture in Europe, which is dominated by high-value meat and dairy production (Eurostat 2020). The study sites were in ten different countries and covered a wide range in terms of severity of COVID-19 outbreak and response strategies (Table 1). For example, Switzerland had a high incidence of COVID-19 cases and deaths in 2020 but a relatively low containment index (composite measure of policy response including school closures, workplace closures, travel bans, testing policy, contact tracing, and face coverings) (Ritchie et al. 2020). Spain, on the other hand, had a high incidence of deaths and a relatively high containment index, whereas Norway had low incidence of COVID-19 cases and deaths and a low containment index (Table 1).

**Table 1:** Study sites, farm characteristics, and COVID-19 severity in 2020. The farm characteristics were derived from the farmer interviews. Farm area and livestock units per farm display median values and median absolute deviations. Data on COVID-19 cases, deaths, and containment were calculated from Our World in Data (Ritchie et al. 2020).

ID	Site	Lat	Long	Farm area [ha]	Livestock units per farm	Most important products	Total COVID-19 cases per million in 2020	Total COVID-19 deaths per million in 2020	COVID-19 containment index for 2020	Summary of local COVID-19 effects on agricultural systems as observed by local co-authors	Nr. of interviewed farmers
CH-1	Reuss, Switzerland	47.23	8.388	29 ± 12	57 ± 35	Milk, pigs, vegetables	51,896	903	43.8	Increased demand for local, especially organic, food products	20
CH-2	Entlebuch, Switzerland	46.95	8.01	18 ± 8	30 ± 26	Milk, pigs	51,896	903	43.8	Increased demand for local, especially organic, food products	21
CH-3	Urserental, Switzerland	46.62	8.57	22 ± 14	19 ± 10	Milk, lambs, cattle	51,896	903	43.8	Increased demand for local, especially organic, food products	19
DE-1	Querfurter Platte, Germany	51.4	11.73	725 ± 649	548 ± 422	Wheat, milk	20,497	394	50.8	The study site is located in a state which had one of the highest COVID-19 incidence rates in the country and experienced prolonged lockdowns, but the severe drought from 2018-2019 overshadowed COVID-19 effects for farmers	15
ES-1	Colmenar Viejo, Spain	40.66	-3.766	150 ± 133	117 ± 80	Cattle	41,250	1088	49.2	Decreased demand for high-quality beef due to the closing of restaurants, markets, and fairs	15
ES-2	St. Maria del Paramo, Spain	42.36	-5.747	52 ± 19	0 ± 0	Corn	41,250	1088	49.2	Little to no effects for arable farming	20
FR-1	Ille-et-Vilaine, France	48.5	-1.564	96 ± 55	106 ± 74	Milk, cattle	39,463	959	51.7	Dairy prices declined while cattle prices increased from 2019. Wheat and corn grain prices increased, but this was due to the drought	16
GR-1	Lemnos, Greece	39.88	25.14	40 ± 45	65 ± 40	Milk, lambs	13,389	467	52.6	Milk prices increased in 2020 but not as a result of the pandemic	19
GR-2	Lesvos, Greece	39	26.47	5 ± 3	0 ± 0	Olives	13,389	467	52.6	Olive oil prices and production were not affected by the pandemic	20
LV-1	Lielvircava, Latvia	56.52	23.75	320 ± 400	0 ± 0	Cereals	21,909	340	43.2	Vegetable and fruit farmers suffered from labor shortages; grain farms were not affected much, except increase in gas and fertilizer prices; dairy farming experienced price drops due to decline in milk export	14
NL-1	Scherpenzeel, Netherlands	52.08	5.49	40 ± 31	182 ± 122	Milk, eggs, pigs	46,970	667	45.5	Most farmers not affected due to price agreements or only minimal effects on price	16
NL-2	Flevopolder, Netherlands	52.43	5.504	70 ± 45	83 ± 122	Vegetables, potatoes, milk	46,970	667	45.5	Disruption of potato demand for fries delivered to restaurants, cafes, and hotels	19
NO-1	Hedmark (Innlandet), Norway	62.42	10.87	38 ± 11	31 ± 32	Cattle, milk	9069	80	36.2	While vegetable and berry farmers suffered from labor shortages, livestock farms were generally not very affected	7
PL-1	Powiat Miechowski, Poland	50.39	20.27	14 ± 7	0 ± 0	Cereals	34,259	755	43.4	Labor shortage because Ukrainian farm workers could not cross the border	20
SK-1	Turzovka, Slovakia	49.41	18.63	25 ± 35	10 ± 13	Milk, hay	50,393	392	49.8	Temporary labor shortage	16

Each site consisted of a roughly 25 km^2^ area representative for the larger region in terms of geography and agricultural practice. Farmers were selected based on their location within the study area (rather than based on a common attribute), so they could be from any of the full diversity of farm types within each study area, reflecting the range of production systems present in most landscapes. In DE-1, LV-1, NO-1, NL-1, NL-2, and SK-1, where it was not possible to find enough interview partners within the study area, the radius for interviews was extended to neighboring farms still operating in a similar landscape.

### Farmer interviews

Overall, 257 interviews were conducted. In each site, the goal was to conduct 15-20 face-to-face interviews, structured around a questionnaire. Interviews were planned for October-November 2020, but during this time the second wave of the pandemic hit, and most interviews had to be postponed. As a result, the interviews were carried out between October 2020 and September 2021. In two sites (DE-1, NO-1), personal visits were not possible, so questionnaires were sent by mail or email after telephone consultation. Also, in NO-1, only 7 interviews could be conducted. All interviews were conducted by persons who spoke the local language and were knowledgeable of local agricultural practices and culture. Prior to data collection, the questionnaires were translated and the lead author and the local partner, if necessary with a translator, went through the questionnaire question by question to reduce the risk of misunderstandings or translation errors. Upon completion of the interviews, data were translated into English and cross-checked by the local contact and the lead author. In case of uncertainties, farmers were re-contacted for clarification. All interviewees provided written consent prior to participating in the study. The experimental design and the questionnaires received ethical clearance from the Ethical Commission of the Swiss Federal Institute of Technology (ETH-EK 2020-N-146), as well as by the relevant authorities in the participating countries where required.

We asked farmers several questions to determine the perceived impact of COVID-19 on farms and different aspects of their livelihoods. First, we asked farmers an open question (“How was your farm impacted by the pandemic?”) to get an overview and place the follow-up questions in context. To make answers comparable, the phrase “in 2020” was added to interviews conducted in the year 2021. We then asked participants to classify the impact as “worst crisis in a lifetime,” “worst crisis in a decade,” “slightly bad year,” “no impact,” or “good year”. Then, we asked participants to approximate the impacts of the pandemic on farm productivity (“amount of farm products that were produced”), sales (“amount of products that could be sold”), received price (“price received for farm products”), labor availability (“labor availability”), and the supply of goods and services (“shortage in supplies and technical support/services”) on a Likert scale from −3 (very negative) to 3 (very positive). The data on overall perceived impact was then related to farm(er) characteristics such as age, farm type, and farm structure, as well as farm specialization, management intensity, and farm size (see Helfenstein et al. (2022) for details on the questionnaire).

### Indicator calculation

We defined a broad set of indicators in order to compare farms from very heterogeneous agricultural systems (Helfenstein et al. 2020). Share of main product, off-farm income, farming system, farm area, age, business type, and farm type were directly assessed through the questionnaire (Table 2). Production diversity was calculated using the Gini-Simpson index for crops and livestock (Eq. 1) (FAO 2019). In Eq. 1, *p* is the proportion of area occupied by crop *i* or the proportion of livestock units attributed to livestock type *i*. Hence, farms with a high diversity and even distribution of crops and or livestock have a high Gini-Simpson index (close to 1), while farms that are highly specialized on one crop and or livestock type have a low Gini-Simpson index (close to 0). For arable, high-value crop, and permanent crop farms, the Gini-Simpson index was calculated for crops only, and for livestock and dairy farms, the Gini-Simpson index was calculated for livestock only. For mixed farms with both livestock and crops, the relative proportion of both crops and livestock was calculated in a combined index by dividing the relative proportions (*p*_*i*_) by two.

**Table 2:** Indicators used to determine farm specialization, management intensity, and farm size. Intensity index and economic farm size are aggregate indicators.

Category	Indicator	Unit	Definition
Specialization	Share of main product	%	Percentage of farm revenue derived from the main product
	Production diversity	-	Gini-Simpson index of crop and livestock diversity (FAO 2019)
	Off-farm income	%	Percentage of farmer’s income derived from off-farm employment
Management intensity	Intensity index	-	Average intensity rank of the following five indicators: N fertilizer use on the main crop, no. of pesticide applications on the main crop, feed import, livestock density, and share of ecological focus area. See the “Methods” section for details
	Farming system	-	Categorical variable to differentiate between certified organic farms and non-organic farms
Size	Farm area	ha	Total area of agricultural land managed by the farm
	Economic farm size	Euros	Approximation of the economic farm size. See the “Methods” section for details
	Megastables	-	Categorical variable differentiating between megastables (> 500 livestock units), large livestock operations (100-500 livestock units), and small livestock operations (< 100 livestock units)
Other	Age	Years	Farmer’s age
	Family farm	-	Categorical variable to differentiate between family farms and corporate farms
	Production type	-	Categorical variable of production types. Farms are classified as either arable, dairy, livestock (pig, chicken, cattle, etc.), permanent crop (fruits and nuts, including olives), high-value crops (vegetables and herbs), or mixed (if more than one category applies)

1 $$ \mathrm{Gini}-\mathrm{Simpson}\ \mathrm{index}\ \mathrm{of}\ \mathrm{diversity}=1-\sum {p}_i^2 $$

To compare management intensity between the wide range of livestock and crop farm types, we introduced an aggregate intensity indicator accounting for nitrogen fertilizer use on the main crop (including from both mineral and organic sources), number of pesticide applications on the main crop, feed import, livestock density, and share of ecological focus area (all derived from the questionnaires). Nitrogen fertilizer use and number of pesticide applications on the main crop together with livestock density are common indicators of land use intensity (Herzog et al. 2006; Geiger et al. 2010; Emmerson et al. 2016). Share of feed import was added since it is an important indicator of input intensity for livestock farms (Helfenstein et al. 2022). Finally, the share of ecological focus area (inverted) was deemed relevant since this is an important indicator for land management intensity (Herzog et al. 2017). The indicator was calculated as the average rank in the overall sample of the five intensity indicators, normalized from 0 to 1. Hence, a value of 0 means the farm ranked the least intensive for all indicators, whereas a value of 1 means the farm ranked the most intensive for all indicators.

Economic farm size [standard output in euros] was approximated based on high-value crop area, crop area, permanent crop area, and number of livestock units following Eq. 2.

2 $$ \log\ (EFS)=0.65\times \log \left(\mathrm{high}-\mathrm{value}\ \mathrm{crop}\ \mathrm{area}+1\right)+0.36\times \log \left(\mathrm{permanent}\ \mathrm{crop}\ \mathrm{area}+1\right)+0.18\times \log \left(\mathrm{crop}\ \mathrm{area}+1\right)+0.71\times \log \left(\mathrm{livestock}\ \mathrm{units}+1\right)+7.90 $$

Equation 2 is derived by fitting a multiple regression model to Eurostat data (Eurostat 2020). The model had a fairly good approximation of average regional farm economic output of farms for Eurostat data (F-statistic = 468, *p* < 0.001, adj. R^2^ = 0.88), and was thus deemed a suitable proxy to approximate economic farm size in our dataset.

A megastable (sensu Debonne et al. (2022)) variable was calculated to differentiate between farms with > 500 livestock units, 100-500 livestock units, and < 100 livestock units.

### Hypothesis testing

We tested the relationship between each numeric indicator and the overall perceived COVID-19 impact using Kruskal-Wallis rank sum tests, a robust alternative to one-way ANOVA (Hollander and Wolfe 1973). If the test result was significant (*p* < 0.05), i.e., at least one group median was different from the group median of another group, a post hoc Dunn test was used for pairwise analysis of difference (Dunn 1964). We tested the relationship between categorical variables and overall perceived COVID-19 impact using cross tabulation and Pearson’s chi-square test (Agresti 2007). Economic farm size and farm area were log-transformed prior to analysis, since these variables had skewed distributions.

### Random forest classification

To complement bivariate hypothesis testing, we fitted a random forest model to determine the relative importance of the different explanatory variables and to study their marginal effect on the response. Random forest classification has been advocated for interview data with Likert-scale response variables, since it does not require assuming mathematical distances between answer options, and is thus more conservative than ANOVA, ordinal logistic regression, or mixed-effects regression models (Endresen and Janda 2016). We collapsed all negative impacts (“worst crisis in a lifetime,” “worst crisis in a decade,” and “slightly bad year”) into one negative class, resulting in a total of three classes for random forest classification: negative impact, no effect, and positive impact. We then used the *randomForest* package in R (R Core Team 2021) and 500 trees with three nodes each for fitting (Liaw and Wiener 2022). Missing explanatory variables were imputed using proximity from randomForest (Liaw and Wiener 2022). Partial dependence was calculated as the marginal effect of one predictor on the response while accounting for the effect of the other predictors (Gareth et al. 2013).

## Results

### Impact of COVID-19 on farms in the 15 case study sites

**Fig. 2:**
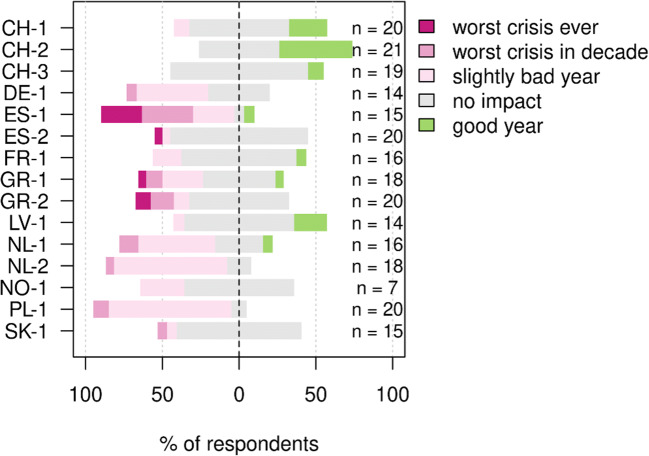
Overall perceived impact of the COVID-19 pandemic on farms in 15 study sites.

Overall, 35% of interviewed farmers reported that they had been negatively affected by the COVID-19 pandemic. In more detail, 3% perceived it as the worst crisis in a lifetime, 7% as the worst crisis in a decade, and 26% as a slightly bad year. A small majority of farmers (55%) reported to be unaffected by COVID-19, and 9% stated that they had an above average year. Impact varied between and within study areas (Fig. 2). Farmers from the Spanish ES-1 site (Colmenar Viejo) reported the strongest impacts. Most farmers in ES-1 produce beef and sell to restaurants in Madrid, which were closed for a large part of the year (Table 1). Several farmers in the region also breed bulls for bullfighting, which was discontinued during the pandemic. All farmers who said it was the worst crisis in a lifetime were located in the four Mediterranean sites. However, within the Mediterranean sites, farmers in ES-2, who mostly practice arable farming, reported to be least affected. Farmers from the Swiss and Latvian study sites (CH-1, CH-2, CH-3, and LV-1) were most likely to have perceived positive effects.

**Fig. 3:**
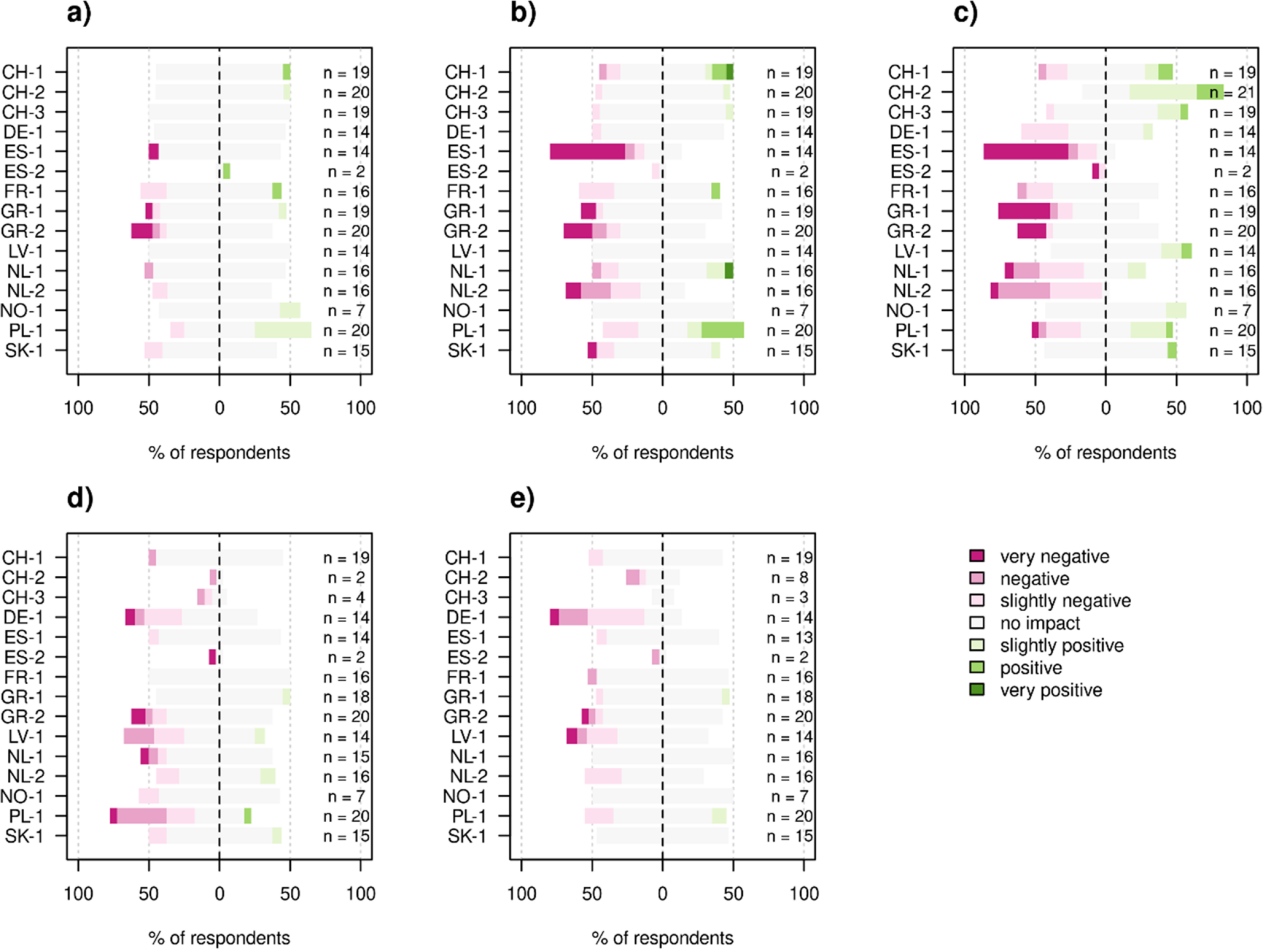
Perceived impact of the COVID-19 pandemic on various aspects of farm functioning.: **a**) Productivity., **b**) Sales., **c**) Price., **d**) Labor availability., and **e**) Availability of supplies and services.

Of the specific livelihood components, farmers most frequently perceived sales and prices to have been affected. Productivity was only affected in 7% of cases (Fig. 3a, sum of all pink area). Severely affected farmers (e.g., in ES-1) mostly suffered from lower sales and lower prices (Fig. 3b and 3c). Similarly, farmers who reported positive effects of the pandemic perceived that these effects were related to either increased sales and/or prices. For example in Switzerland, farmers who sell directly to consumers benefited from increased demand and/or achieved better prices through direct sales. Labor availability was an issue in several study sites, most notably in the Polish PL-1 site (Fig. 3d). In PL-1, farmers are dependent on Ukrainian farm workers, who were not able to cross the border into Poland during the lockdown (Table 1). Shortage of supplies and services also affected 18% of farms, especially in DE-1, where lockdowns were particularly long and most farms were large corporations dependent on complex and vertical supply chains (Fig. 3e). The responses to the questions on specific livelihood components correlated well with overall perceived impact (*χ*2 = 99.2, *p* < 0.001, Supplementary Fig. 1a). Also, analysis between individual livelihood components and overall perceived impact confirm that perceived effect on sales and prices match most closely with overall perceived impacts (Supplementary Fig. 1b-f).

### Production type, farmer characteristics, and business type

**Fig. 4:**
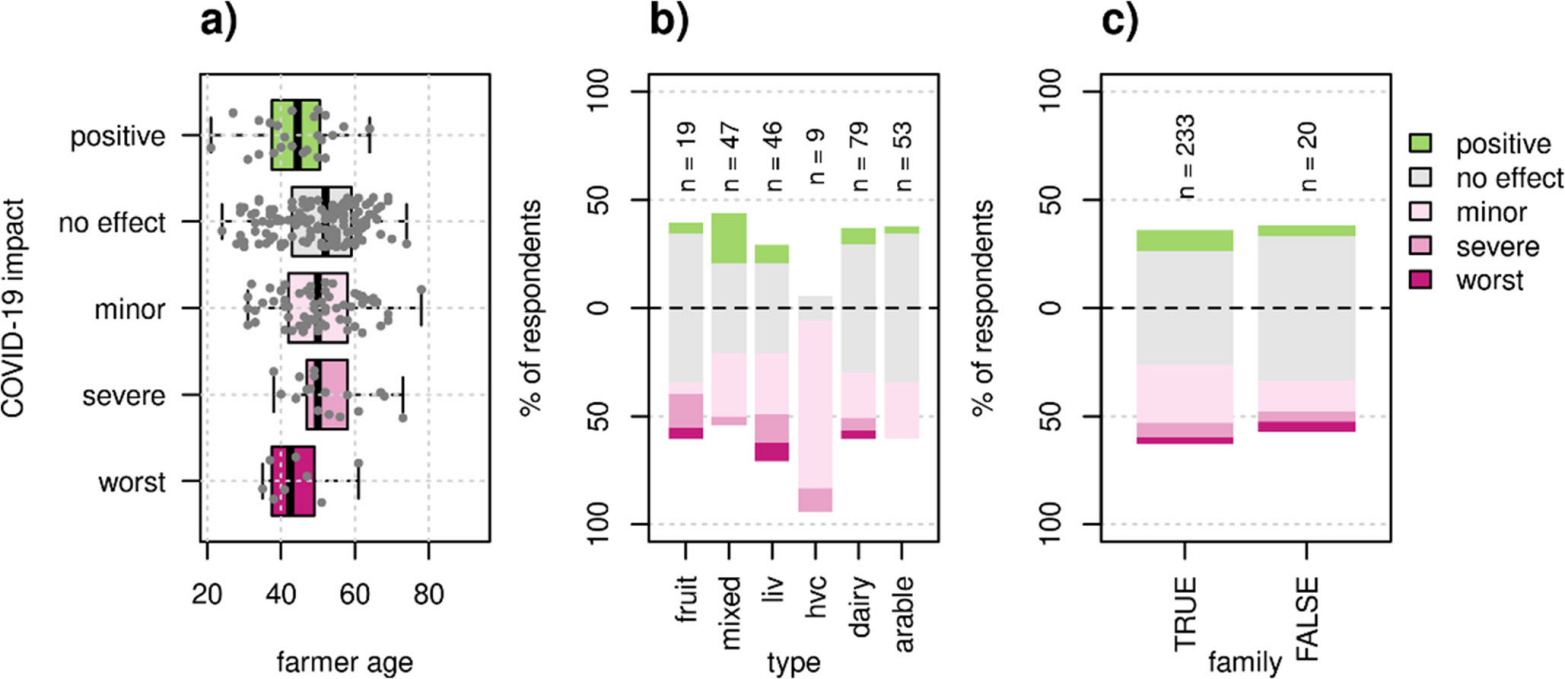
Relationship of farmer age, production type, and family farms with perceived COVID impact. Though farmer age (**a**) significantly differed between groups according to a Kruskal-Wallis test (χ2 = 10.7, p = 0.03), none of the groups were significantly different according to a post -hoc Dunn test. Each dot represents one farm. Production type (**b**) significantly affected COVID impact (χ2 = 53.2 (20), p < 0.001). Production type “fruit” includes fruit and olive farmers, “liv” stands for non-dairy livestock farms, and “hvc” stands for high-value crop farms. There was no significant effect of family farms (**c**) (χ2 = 2.7 (4), p = 0.60).

Farmer age showed a significant relationship with perceived COVID-19 impact (*χ*2 = 10.7, *p* = 0.03), with most affected and positively affected farmers appearing to be younger than farmers from the other classes, but post hoc Dunn test pairwise tests were not significant (Fig. 4a). Farm type had a strong effect on perceived COVID-19 impact (Fig. 4b). High-value crop farms were most likely to perceive negative effects, with 89% of farms reporting some sort of negative effect, followed by livestock farms with 50%. Arable farms were least likely to perceive negative effects, as 70% of arable farms reported “no effect”. Twice as many mixed farms perceived to benefit from the pandemic compared to all other farm types (Fig. 4b). If the farm was a family farm or not did not affect COVID-19 impact (Fig. 4c).

### Farm specialization

**Fig. 5:**
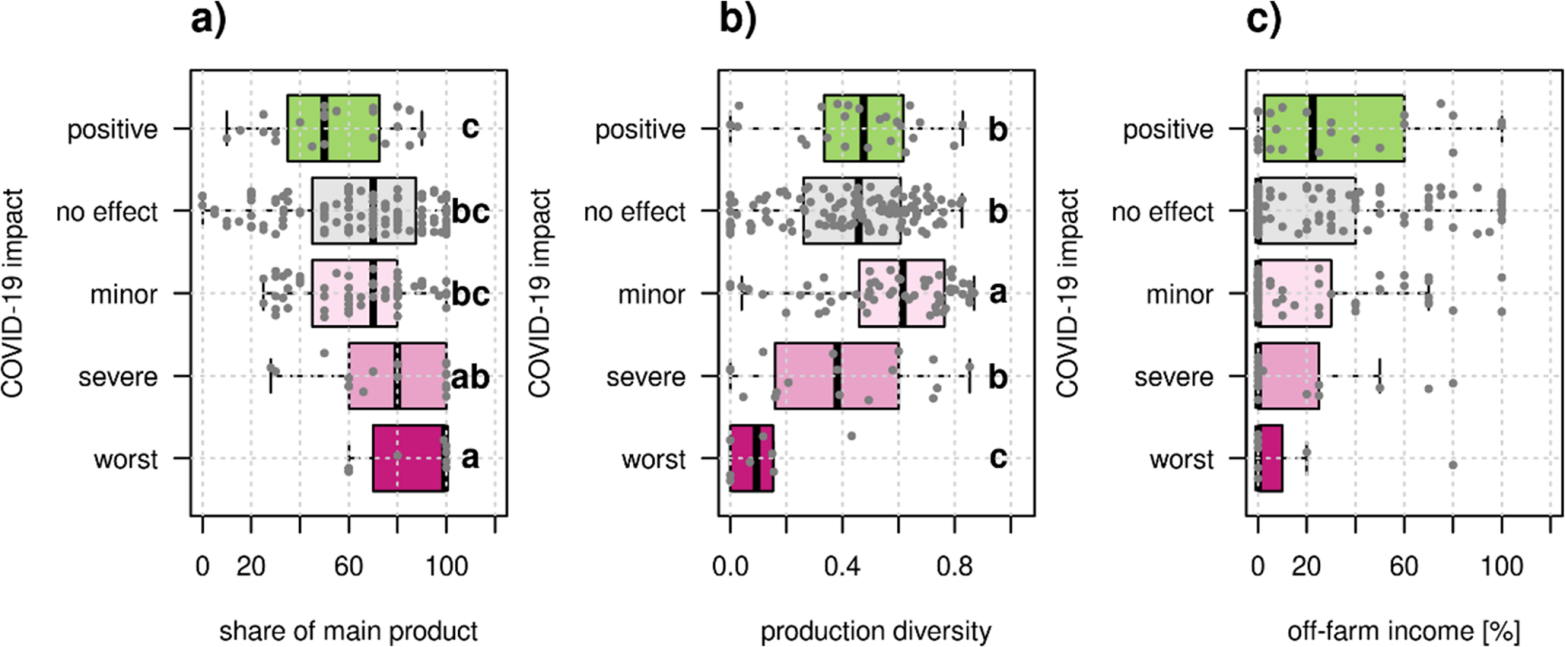
Relationship between farm attributes related to farm specialization and perceived COVID-19 impact. The share of revenue attributed to the main farm product (**a**) (χ2 = 14.2, p = 0.007) and production diversity (χ2 = 30.5, p < 0.001) significantly affected COVID-19 impact. The share of off-farm income (**c**), on the other hand, did not significantly affect COVID-19 impact (χ2 = 8.0, p = 0.09). All Kruskal-Wallis tests had 4 degrees of freedom. Different letters show significant differences between groups according to post -hoc Dunn tests. Each dot represents one farm.

Farms with high reliance on one product perceived to be the most affected by the COVID-19 pandemic. The median share of the main product was 99.5% for farmers who reported that the pandemic was the “worst crisis in a lifetime,” and decreased gradually to 50% for farmers who reported benefiting from the pandemic (Fig. 5a). Also, farmers who were affected worst had the lowest median production diversity (Fig. 5b). However, the group with minor negative effects had the highest production diversity. We observed a trend that farms that perceived to be less affected by COVID-19 had a higher fraction of income from off-farm employment (Fig. 5c), but this was not statistically significant. Overall, farmers of the class “worst crisis in a lifetime” were highly specialized according to all three indicators. They had the highest median main product share, lowest median production diversity, and lowest share of off-farm income. While some farmers that perceived “no effect” or “positive effect” also had high degrees of specialization, farmers from those groups were on average more diversified than farmers from the groups “worst crisis in a decade” and “worst crisis in a lifetime”.

### Farm intensity

**Fig. 6:**
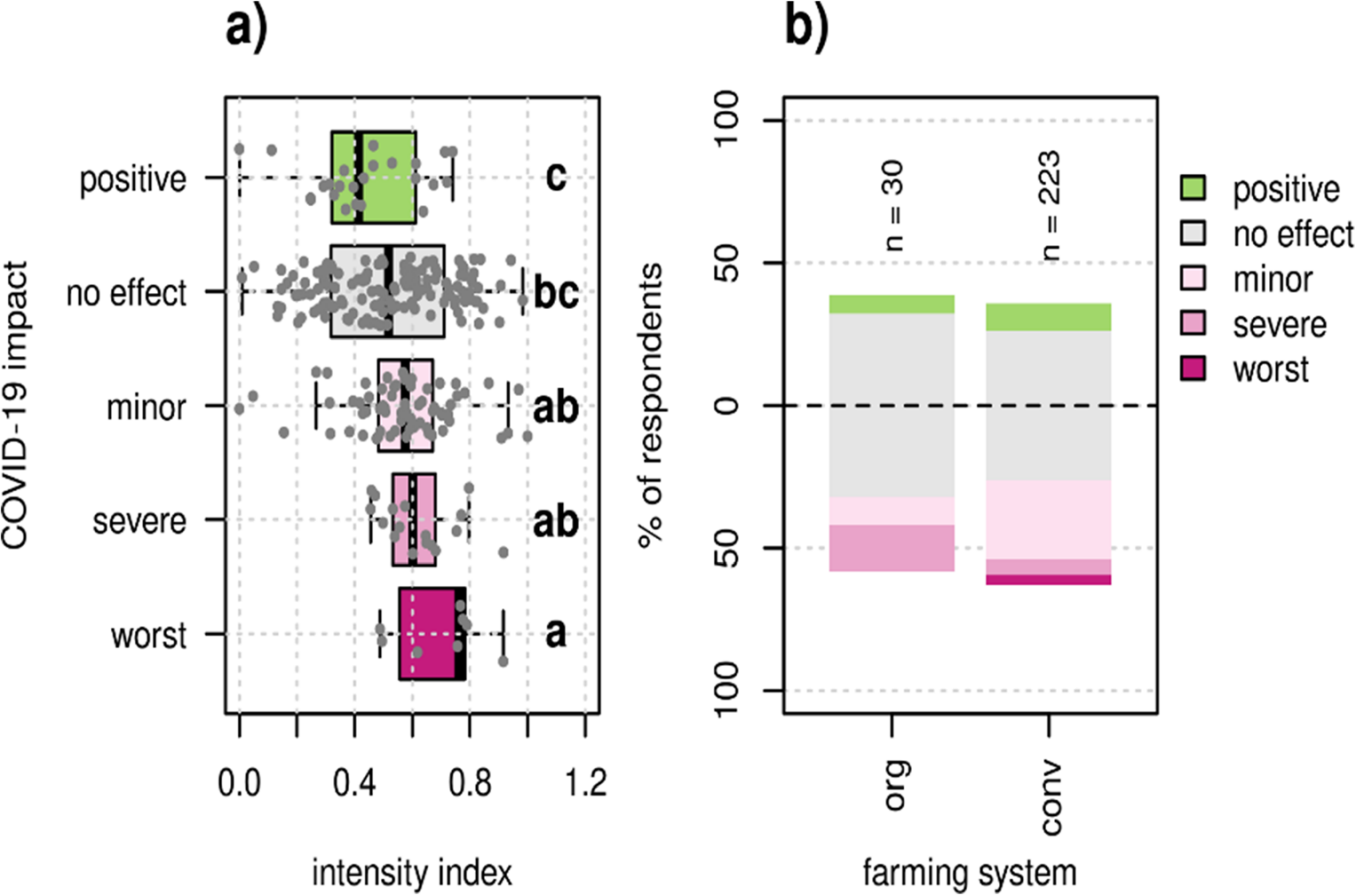
Relationship between farm attributes related to management intensity and perceived COVID-19 impact. The intensity index (**a**) significantly affected COVID-19 impact (χ2 = 15.9, p = 0.003), according to a Kruskal-Wallis test with 4 degrees of freedom. Different letters show significant differences between groups according to a post -hoc Dunn test. Each dot represents one farm. The farming system (**b**) also significantly affected COVID-19 impact (χ2 = 10.7 (4), p = 0.041), according to a Pearson’s chi-squared test. org: organic farming; conv: non-organic farming.

We found that intensively managed farms more often reported to have been negatively affected by the pandemic (*χ*2 = 15.9, *p* = 0.003). The most severely affected group had the highest median intensity and farms that benefited had the lowest median intensity (Fig. 6a). The farming system itself was also significant (*χ*2 = 10.7 (4), *p* = 0.041). More non-organic farms experienced the worst crisis in a lifetime or a slightly bad year, while more organic farms experienced the worst crisis in a decade. Non-organic farms on average had a higher intensity than organic farms (Wilcoxon test = 4657, *p* = 0.003) (Supplementary Fig. 2).

### Farm size

**Fig. 7:**
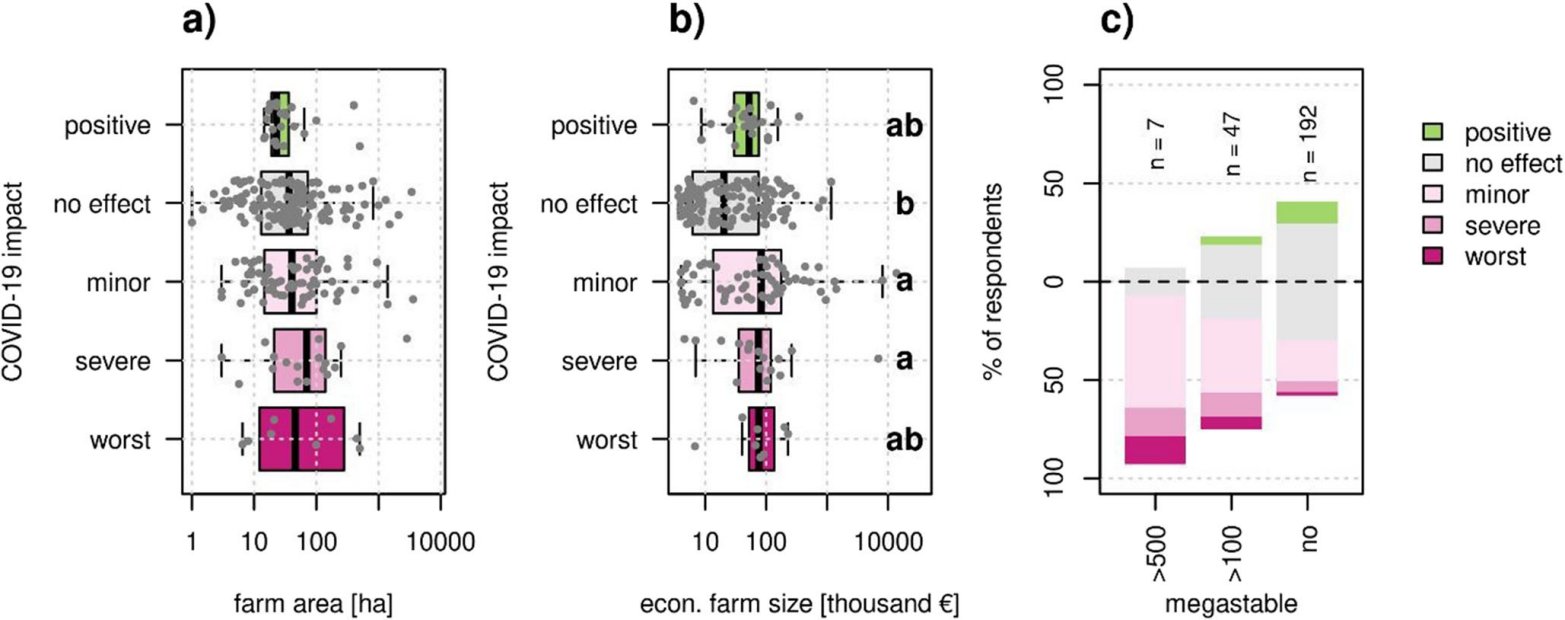
Relationship between farm attributes related to farm size and perceived COVID-19 impact. Farm area (**a**) did not significantly affect COVID-19 impact (χ2 = 3.8, p = 0.44), while economic farm size (**b**) did significantly affect COVID-19 impact (χ2 = 18.3, p = 0.001). Both Kruskal-Wallis tests had 4 degrees of freedom. Different letters show significant differences between groups according to a post -hoc Dunn test. Each dot represents one farm. Megastables (> 500 livestock units) and large livestock farms (100--500 livestock units) were more likely to have been negatively affected by COVID-19 (χ2 = 23.6 (8), p = 0.001), according to a Pearson’s chi-squared test (**c**).

We found no relationship between farm area and perceived COVID-19 impact (Fig. 7a). However, farms that were not affected by COVID-19 had smaller economic farm size than those that were negatively affected (Fig. 7b). Also, megastables (> 500 livestock units) were more negatively affected than large livestock farms (100-500 LU), and small livestock farms (< 100 LU) were least affected (Fig. 7c).

### Random forest classification

The random forest classification model with all explanatory variables (Table 2) had an overall out-of-bag estimate of error of 24.5%. The confusion matrix (Supplementary Table 1) shows that misclassification was more likely for positive perceived COVID-19 impact (75%), and much lower for negative impact (25.3%) and no effect (15.2%). Hence, the random forest model was informative for studying farm attributes that were associated with a perceived negative effect, but less accurate to explain why some farmers benefited from the pandemic.

**Fig. 8:**
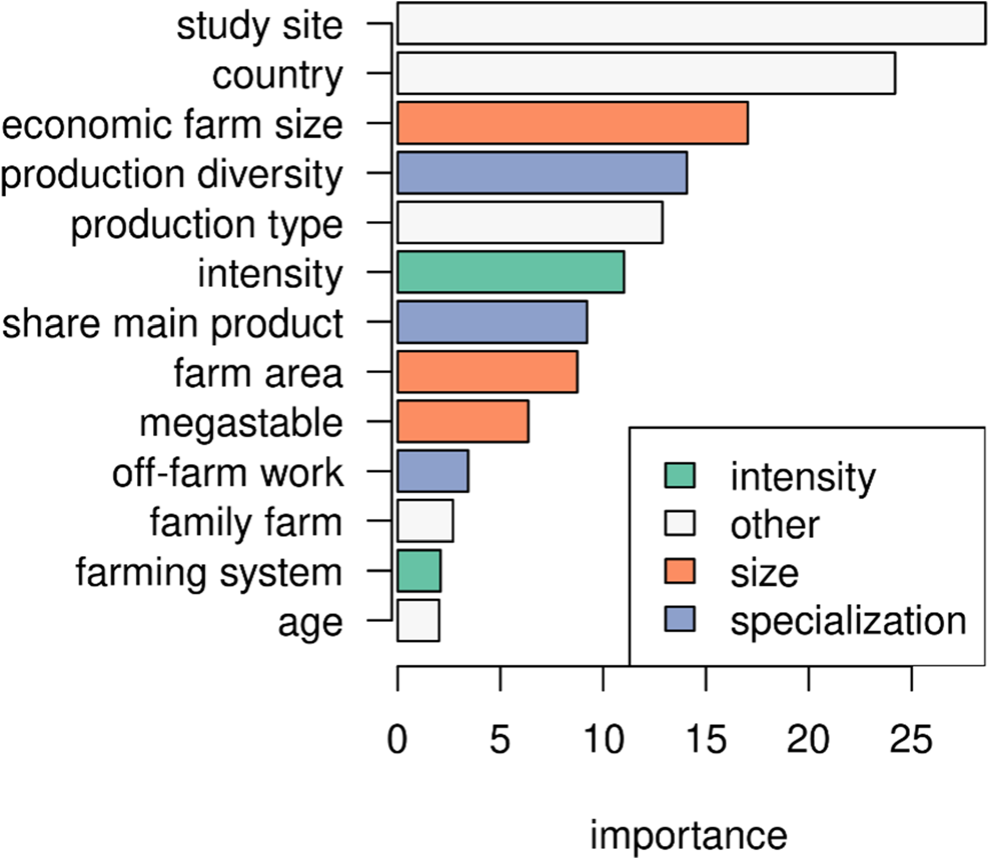
Relative importances for predicting perceived impact of the COVID-19 pandemic. The relative importance of a variable, here mean decrease in accuracy, is calculated by looking at how much prediction error increases when data for that variable is permuted while all others are left unchanged (Liaw and Wiener, 2022).

Study site and country were the most important explanatory variables (Fig. 8), followed by economic farm size, production diversity, production type, and management intensity. Off-farm work, family farm, farm type, and farmer age had the lowest relative importance to predict perceived COVID-19 impacts. These results largely confirm those of the bivariate hypothesis testing, i.e., variables that had a significant effect on perceived COVID-19 impact in bivariate testing generally had a higher relative importance in the random forest classification model. Exceptions were farm area and farming system. Farm area was not a significant predictor in the bivariate test while farming system was; in the random forest model, the former was more influential than the latter. These differences are because in the random forest model, all negative impacts were grouped into one class. For the farming system, the significant difference in the bivariate testing was between different severity classes, which were not accounted for in the random forest model.

Partial dependence plots give a more nuanced view of the impact of farm attributes on COVID-19 impact by showing the marginal effect of a given variable while accounting for the other variables (Supplementary Fig. 3). The partial dependence plots largely confirm the bivariate testing, in that a perceived negative COVID-19 impact was more likely for more intensive farms (Supplementary Fig. 3c) and farms with higher share of main product (Supplementary Fig. 9d). On the other hand, economic farm size and production diversity have more complex relationships with perceived impact (Supplementary Fig. 3a, b). Regarding economic farm size, the partial dependence plot suggests that the smallest farms were the most likely to suffer a negative COVID-19 effect, while medium-sized farms were least likely (Supplementary Fig. 3a). For production diversity, it appears that farms with higher production diversity were actually likely to perceive a negative COVID-19 effect.

## Discussion

**Fig. 9:**
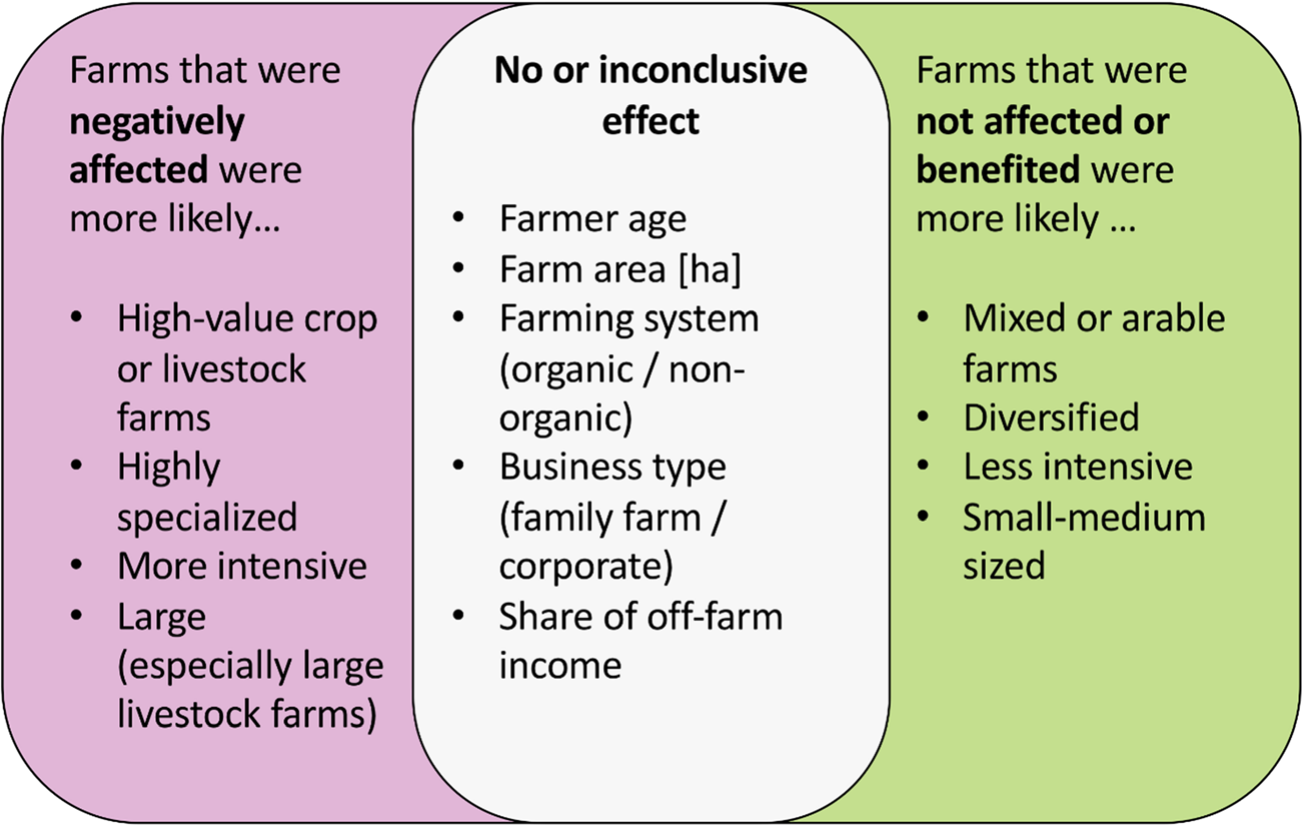
Farm attributes associated with farms that perceived to be affected and farms that did not perceive to be affected by the COVID-19 pandemic, as well as variables that had no or an inconclusive relationship with perceived impact.

To improve our understanding of how the agricultural sector was affected by the COVID-19 pandemic, how farmers perceived those impacts, and which factors explained the severity of impacts is key for addressing major sustainability challenges and for improving farm-level resilience to future shocks. Here, we analyzed the perceived impact of the COVID-19 pandemic on farms from 15 distinct agricultural systems across Europe for the year 2020 using interview data and statistical analyses, which resulted in three key insights. First, our results on farmers’ perceived impacts show that most farmers were not or only slightly affected by the first wave(s) of the pandemic in 2020, confirming objective economic indicators (Montanari et al. 2021). Second, our case study approach showed that COVID-19 impacts were largely dependent on study site characteristics, due to locally variable susceptibilities and responses to the pandemic, as well as cultural differences in subjective evaluations. For example, farmers in ES-1 suffered more than in other study sites from reduced touristic and catering activity due to the pandemic. This echoes the findings of Meuwissen et al. (2021) that local to national level organization, networks, and government support were critical factors explaining the severity of the pandemic on farms across Europe. Third, our study showed that there was also considerable variation in perceived impact even within study sites (Fig. 9). In addition to the well-known effect of farm type and sector (Montanari et al. 2021), variability in perceived impact severity was associated with farm attributes related to farm specialization, management intensity, and farm size. Supplementing traditional hypothesis testing (Kruskal-Wallis test and Pearson’s chi-squared test) with random forest classification allowed disentangling the study site effect from that of other farm attributes. Since the first and second points have been discussed in detail in the references cited above, we will focus the discussion on the third insight.

### Highly specialized farms were more likely to have perceived negative impact

For both crop (Gaudin et al. 2015; Zampieri et al. 2020) and livestock (Dumont et al. 2020) systems, diversity has been argued to increase farm performance and resilience. Furthermore, diversity emerged as the central theme for building resilience at the 2021 United Nations food summit (Hertel et al. 2021). Since resilience is a complex and often theoretical concept, more real-world data on benefits of diversification for farms is needed. Our study adds to the mounting evidence that farms with low-level of diversification were more affected by the COVID-19 pandemic. Diversity is also often related with modularity, since if one income source fails, farmers may still fall back on other sources of income. For example, an analysis of the pandemic’s impact on Flemish (Belgium) farmers showed that diversity of production processes and marketing channels increased both coping and responsive capacities (Coopmans et al. 2021). In our analysis across Europe, we considered three indicators of farm diversification. The clearest relationship was observed for the share of revenue derived from the main product (Fig. 5a, Supplementary Fig. 3d), a strong proxy for farm specialization. If the share of main product is high, this means either that production is heavily specialized and/or that revenue is mainly dependent on one product. Similarly, we saw that mixed farms (Fig. 4b) were the most likely to benefit from the pandemic. The partial dependence plot of production diversity suggested that farms with higher production diversity were more likely to have experienced some sort of negative effect by COVID-19 (Supplementary Fig. 3b). This may seem counterintuitive at first. However, the finding supports the theory that diversification is an insurance against risk. If a farm is highly diversified, it is likely that one of the products will be affected by an unexpected shock, leading to a slight (but bearable) negative effect. However, bivariate testing showed clearly that severe impact was significantly less likely for diversified farms (Fig. 5b). In other words, farmers that placed their eggs in several baskets were likely to lose one egg; however, they were insured against losing all their eggs.

Our results support that in an increasingly uncertain world, a certain level of diversity is key to maintaining farmers’ livelihoods. However, in practice, farmers often face a trade-off between efficiency and diversity and or lock-ins that hinder diversification (Coopmans et al. 2021). Levers that encourage farmers to diversify their production include adaptation of standards and labeling, coordination between stakeholders to fairly share added value within value chains, and genetic, agronomic, technological, and organizational innovations (Meynard et al. 2018). Specifically, future technological development should focus on accommodating more diverse agricultural systems (e.g., robotics for intercrops), rather than promoting further simplification (Ditzler and Driessen 2022).

### Intensive farms were more likely to have perceived negative impact

Several authors were quick to use the COVID-19 pandemic to promote a transition to agroecology, however without providing empirical evidence (Altieri and Nicholls 2020; Loker and Francis 2020). In our study, we found that perceived adverse COVID-19 impacts were more likely for farms with a high intensity score (Fig. 6a, Supplementary Fig. 3b). It is possible that intensive farms had stronger integration in value chains, making them more sensitive to supply chain disruptions; were more dependent on migrant labor and thus more affected by closed borders; exhibited high use of consumable inputs (fixed costs), making them more sensitive to changes in price and demand; and operated with low margins and were thus more sensitive to price changes (Meuwissen et al. 2021; Petersen-Rockney et al. 2021). The abovementioned possible explanations have to do with tightness of feedbacks, an important resilience enhancing attribute (Meuwissen et al. 2019). For example, in ES-1, where the share of imported feed is high, some farmers mentioned that the high fixed cost for feed was in contrast to fluctuating prices for the meat. However, these are but a list of possible explanations, which could not be tested further. The fact that both specialization and management intensity correlated with COVID-19 impact supports the notion that agroecological transition is a viable pathway to increase resilience to future unforeseen shocks (Tittonell et al. 2021). In that sense, our paper adds empirical evidence to earlier claims made on more theoretical grounds.

### Large farms were more likely to have perceived negative impact

While farm area per se was not a meaningful indicator for COVID-19 impact (Fig. 7a), a proxy of economic farm size, considering not only the area but also the type of crops and the livestock units, was a significant predictor (Fig. 7b). Our results show that larger farms were more likely to be negatively affected. The exception to this general trend is that of the smallest farms, though, which were the most likely to be negatively affected (Supplementary Fig. 3a). We can explain this tendency using the concept of flexibility, which has also been argued to be important for explaining farm adaptive and coping capacity in the COVID-19 pandemic (Coopmans et al. 2021; Meuwissen et al. 2021; Tougeron and Hance 2021). The smallest farms tend to be less flexible because they lack reserves to deal with shocks. On the other hand, large farms usually are more dependent on the global market, both for inputs and selling their products, which also make them vulnerable to economic and trade shocks such as the 2020 lockdown(s). Furthermore, the large production volumes reduce flexibility to respond to unexpected changes. For example, one farmer noted that “less was sold in large quantities for mass food preparation,” while several farmers reported “increased demand for local products” and one said, “life was slower, appreciation for regional food and small-scale farmers increased”.

The challenge for large farms in dealing with unexpected events was especially apparent for livestock farms, where impact severity was higher for megastables and large livestock operations than for smaller farms (Fig. 7c). Several farmers operating large livestock farms mentioned increasing price for feed. It is likely that large livestock operations have higher fixed costs (e.g., due to contract agreements for inputs and services, depreciation, interest payments, maintenance costs), and therefore their economic performance is more sensitive to sudden changes in demand and prices (McEwan et al. 2020; Thorsøe et al. 2020). Arable farms in comparison often have cheaper inputs and do not have perishable products, giving them more flexibility to wait until supply chains recover and or prices improve. Hence, arable farms were least affected by the COVID-19 pandemic (Fig. 4b) and farm area did not increase perceived COVID-19 impact (Supplementary Fig. 3e).

### Implications for future unforeseen shocks

The past few years have made it painfully clear that the world has become less predictable. Extreme weather events, the COVID-19 pandemic, and geopolitical confrontation have all affected agricultural systems worldwide. The Global Risk Perception Survey from 2022, conducted by the World Economic Forum with risk experts and world leaders in business, government, and civil society, summarizes that most respondents “expect the next three years to be characterized by either consistent volatility and multiple surprises or fractured trajectories” (WEF 2022). The participants identified the following as most severe risks for the next 10 years: climate action failure, extreme weather, biodiversity loss, social cohesion erosion, livelihood crises, infectious diseases, human environment damage, natural resource crises, debt crises, and geoeconomic confrontation. While different types of shocks affect agricultural systems in different ways, in our globalized food system, a main mechanism impacting farmers is often sudden changes in prices for inputs and products, and disruptions of supply chains. The farmers surveyed in our study confirmed that they perceived changes in prices and sales volumes as a result of the pandemic to affect them most strongly. While the COVID-19 pandemic was a truly global crisis, even regional disasters such as extreme weather or war can disrupt supply chains and affect farmers globally. For example, the 2011 winter drought in eastern China led to a doubling of global wheat prices (Sternberg 2012). Meanwhile, the war in Ukraine is increasing prices of fertilizer, petrol, electricity, and animal feed—raising the costs of production for farmers worldwide.

Resilience theory emphasizes that diversity, openness, tightness of feedbacks, system reserves, and modularity enhance farming system resilience (Meuwissen et al. 2019). Our study confirmed that farmers operating more diversified, less intensive, and (at least for livestock farms) smaller farms perceived to have been less negatively affected by the COVID-19 pandemic. While the impact of any particular future disturbance on individual farms will depend on geography, political context, duration, and the type of disturbance (Debonne et al. 2022), increasing resilience to price shocks through diversification and reducing dependency on external inputs by tightening feedback cycles will likely help farmers deal with a number of different types of shocks.

### Limitations and research outlook

While our results provide valuable insights on European farmers’ perceived exposure to the pandemic and have a broad coverage across important farming regions of Europe, the study has several limitations that need to be considered when interpreting the results. First of all, our study is not representative for all European farmers. The sites cover a large gradient in farming systems and farm types, yet several farm types (e.g., viticulture, which was one of the hardest hit agricultural sectors (Montanari et al. 2021)) were not represented. Also, the sample size was relatively low considering the large spatial extent of our study area. Second, our study focused on subjective resilience. Future studies should compare farmers’ perceived impacts with objective impacts as measured by farm economic data. Third, our study focused on one resilience capacity, namely robustness, but did not cover the other two: adaptability and transformability (Meuwissen et al. 2019). Adaptability and transformability require more time to be detected. To fully understand farm-level resilience to the COVID-19 pandemic, it will be necessary to observe changes to farm structure and management in response to the pandemic in the coming years.

Another limitation of our work is that not all interviews could be conducted in 2020. Though farmers were asked about COVID-19 impacts in 2020, it is possible that answers provided by farmers in 2021 were influenced by their experiences in 2021. Nevertheless, given the considerable diversity of farms included and the significant effects found, the results provide strong evidence for the existence of more generic relations that hold across Europe. At the same time, the exact functional forms of the partial dependence plots should not be over-interpreted, given the relatively low sample size (Supplementary Fig. 3). The predictive power of the random forest model could have been improved if data for other variables were available, such as on supply chains (Meuwissen et al. 2021), and more information on farmer attitudes and motivations (Spiegel et al. 2021). Also, results from the two modes of analysis should be treated as complementary rather than allowing a direct comparison, since the negative COVID-19 impact classes (“worst crisis in a lifetime,” “worst crisis in a decade,” and “slightly bad year”) were grouped into one class for the random forest classification.

## Conclusions

Our study provides insights on subjective resilience to the COVID-19 pandemic, as perceived by farmers. The findings largely mirror objective data on economic impacts, by showing that most arable farmers were not or only slightly affected, while more labor-intensive farms or farms producing perishable items (vegetables, meat, dairy) tended to be more affected. Mainly, perceived impacts were due to disruptions of value chains. In addition, our analysis of relationships between farm attributes and perceived impact revealed that more specialized and more intensive farms were more likely to have perceived negative impacts. Farm size played an important role for livestock farms (but not for arable farms); large livestock farms were more likely to have reported negative effects of the COVID-19 pandemic. These results suggest that future shocks disrupting regional to global supply chains are also likely to hit those farms hardest that are most specialized and highly dependent on external inputs. From a societal perspective, this suggests that ongoing concentration of food production in fewer, larger, more specialized, and more intensive farms bears considerable risk in terms of farmer vulnerability to unforeseen shocks.

## Supplementary Information

ESM 1 (DOCX 612 kb)

## Data Availability

The dataset generated during the current study are available from the corresponding author on reasonable request.
