# Supplementary material for: Farmer surveys in Europe suggest that specialized, intensive farms were more likely to perceive negative impacts from COVID-19
Source: Agron Sustain Dev. 2022 Aug 23;42(5):84. doi: 10.1007/s13593-022-00820-5 (PMC9397162; doi:10.1007/s13593-022-00820-5)
Supplement: Supplementary file 1 — (DOCX 612 kb) [file 13593_2022_820_MOESM1_ESM.docx]

Supplement for Helfenstein et al.

**Supplementary Tables**

Table 1. Confusion matrix of the random forest classification model. The overall out-of-bag estimate of error was 24.5%.

|  | negative | no effect | positive | class error (%) |
| --- | --- | --- | --- | --- |
| negative | 68 | 22 | 1 | 25.3 |
| no effect | 17 | 117 | 4 | 15.2 |
| positive | 2 | 16 | 6 | 75.0 |

**Supplementary Figures**

**
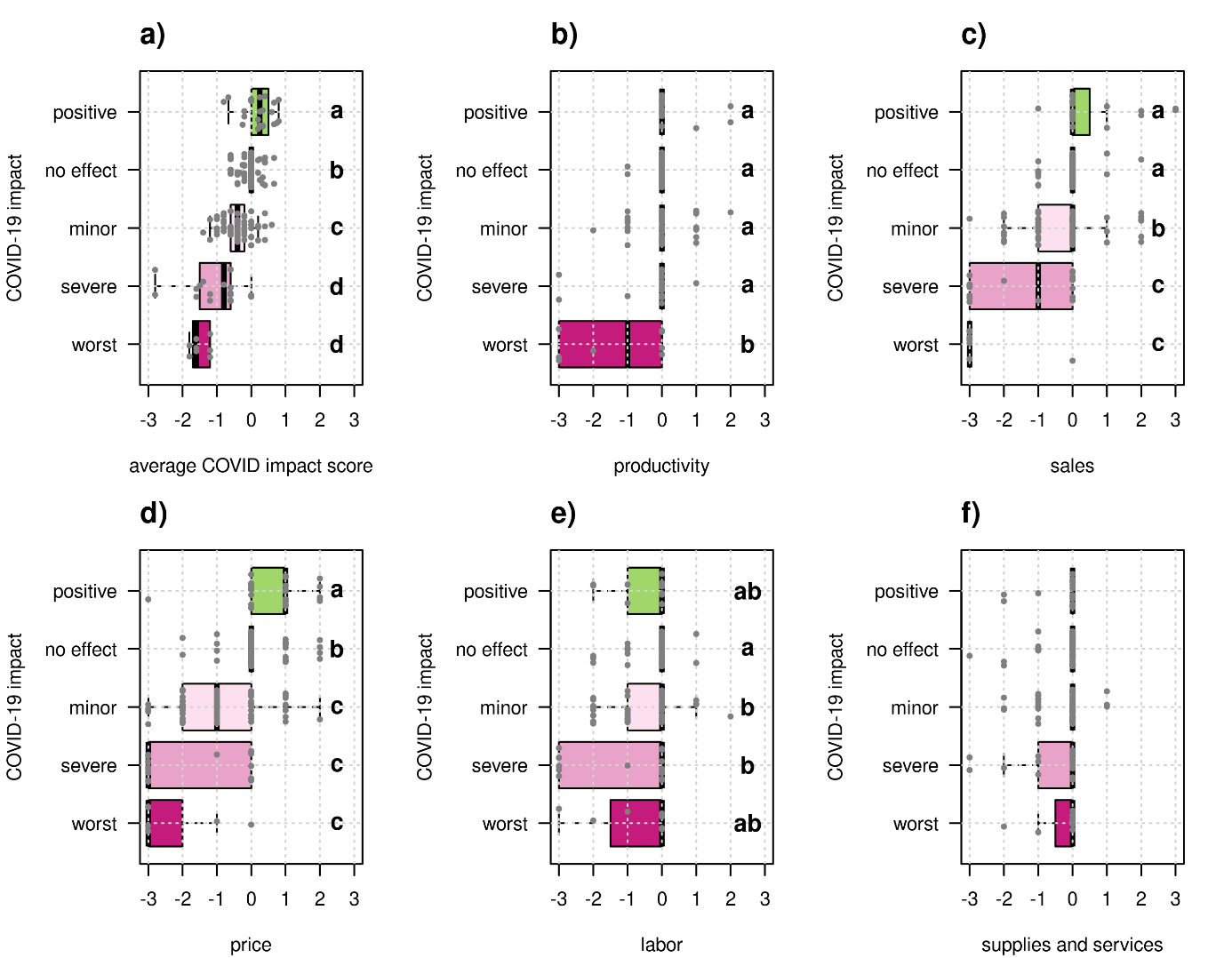
**

Figure 1. Relationship between perceived COVID-19 effect on various aspects of farm functioning and overall impact. For this consistency check, likert-scale answers were treated as numeric. Average COVID impact score (average likert score from productivity, sales, price, labor, and supplies and services) was strongly related to overall perceived impact (a, χ^2^ = 99.2, p < 0.001). A significant relationship was also observed between productivity (b, χ^2^ = 21.7, p < 0.001), sales (c, χ^2^ = 56.7, p < 0.001), price (d, χ^2^ = 56.7, p < 0.001), and labor (e, χ^2^ = 15.8, p = 0.003) and overall perceived impact. There was not a significant relationship between supplies and services and overall impact (f, χ^2^ = 7.6, p = 0.11). All χ^2^ test scores and p-values refer to Kruskal-Wallis tests with four degrees of freedom. Different letters show significant differences between groups according to post-hoc Dunn tests. Each dot represents one farm.


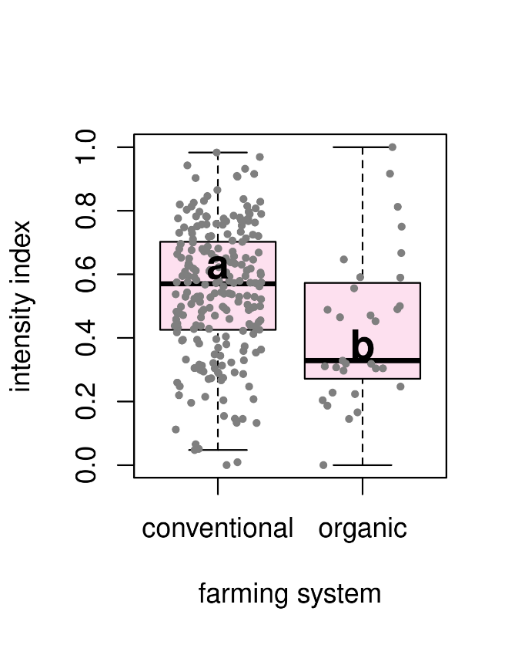


Figure 2. Intensity index as a function of the farming system. Conventional farms (n = 226) had a higher intensity than organic farms (n = 31). Wilcoxon-test = 4657, p = 0.003.


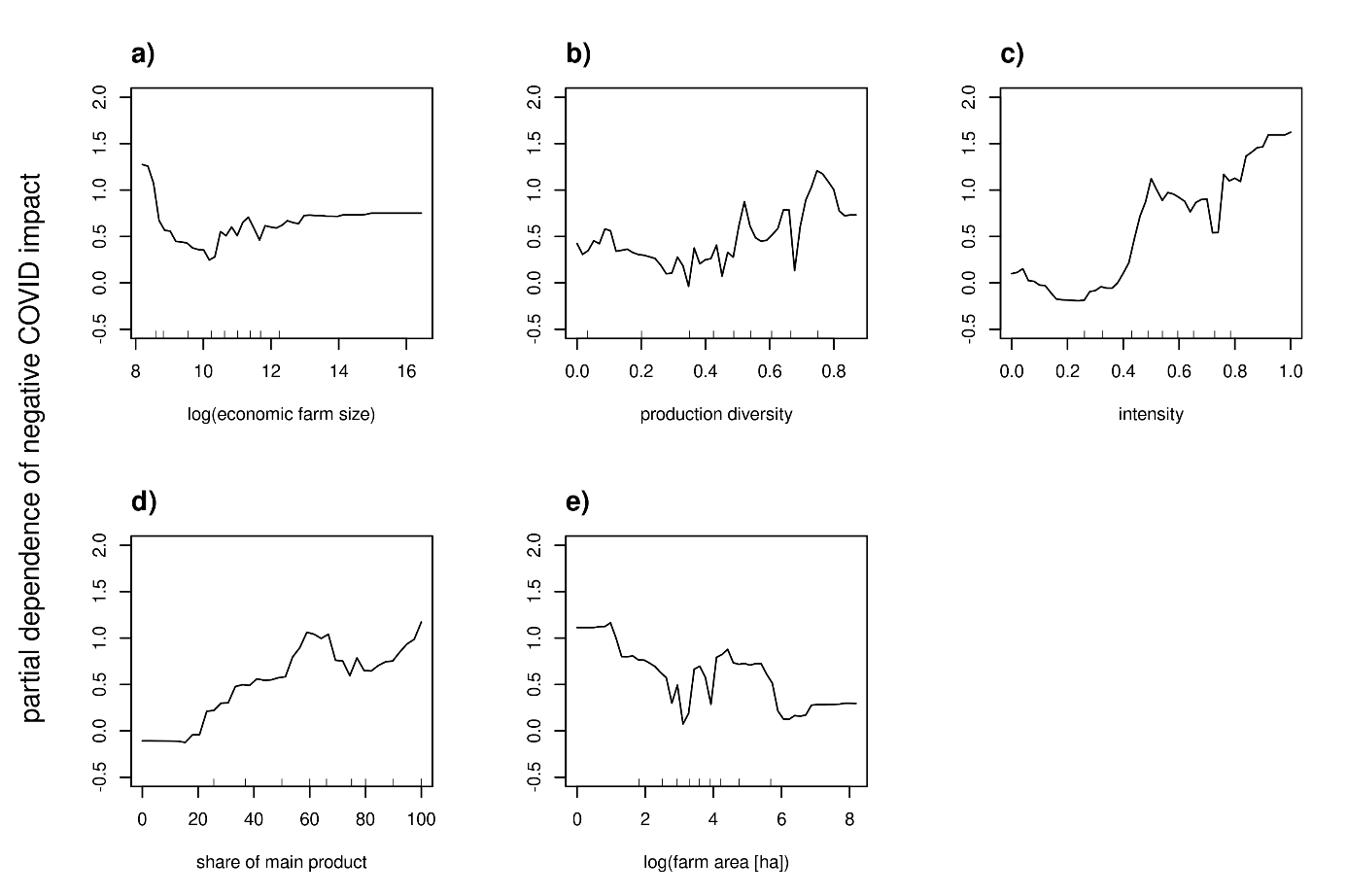


*Figure 3. Partial dependence plots. Partial dependence plots show the marginal effect of one explanatory variable on the response while accounting for the effect of the other explanatory variables. The higher the partial dependence, the more likely a farm with those characteristics experienced a negative impact of COVID-19. a) log(economic farm size), b) production diversity, c) intensity, d) share of main product, and e) log(farm area). The tick marks on the x axis show the decimals of the data distribution.*
